# Homonymous hemianopia due to cerebral venous thrombosis: A case report

**DOI:** 10.1097/MD.0000000000036204

**Published:** 2023-12-29

**Authors:** Woo Seok Choi, Sook Hyun Yoon, Donghun Lee

**Affiliations:** a Department of Ophthalmology, Daegu Catholic University School of Medicine, Daegu, Korea.

**Keywords:** case report, cerebral venous thrombosis, homonymous hemianopia, visual field defects

## Abstract

**Rationale:**

Diagnosing cerebral venous thrombosis (CVT) can be difficult because of nonspecific symptoms, such as headache and homonymous hemianopia (HH). Herein, we present a case of delayed CVT diagnosis due to nonspecific neurological symptoms and nonprominent lesions in a patient with HH.

**Patient concern:**

A 65-year-old woman presented with a sudden onset headache accompanied by right HH that lasted for 1 day. Brain computed tomography and magnetic resonance imaging were initially performed due to suspicion of ischemic lesions or hemorrhage in the left postchiasmal visual pathway; however, no remarkable acute brain lesions were detected. Ophthalmological examinations revealed no notable findings, except for a definite field defect in the Humphrey visual field test. The headaches then waxed and waned but recurred 3 days after the initial symptom.

A repeat brain magnetic resonance imaging was performed, which revealed left sectoral gyral swelling and vascular enhancement in the occipital lobe. To further evaluate venous drainage, additional 3-dimensional cerebral computed tomography angiography and 4-vessel angiography were conducted, revealing a partial filling defect in the left transverse sinus and superior venous drainage impairment. These findings suggested the presence of venous thrombosis in the left transverse sinus.

**Diagnosis:**

The patient was diagnosed with thrombosis of the left transverse sinus, which subsequently caused the right HH.

**Intervention:**

Anticoagulation therapy with parenteral heparin was started as soon as the diagnosis of CVT was confirmed. Eventually, the patient was solely managed with oral warfarin administration.

**Outcomes:**

Following 3 days of treatment, her headache resolved, and a subsequent visual field testing conducted 2 weeks later revealed a definite improvement in the field defect.

**Lessons:**

Despite its favorable prognosis, CVT can be challenging to diagnose. CVT should be considered as a differential diagnosis when diagnosing patients who present with headaches accompanied by HH without prominent brain lesions.

## 1. Introduction

Homonymous hemianopia (HH) is a vision loss in the same meridian half of the visual field in both eyes.^[1]^ HH can occur in any lesion along the contralateral postchiasmal area of the visual pathway and most lesions involve the occipital lobes, followed by the optic radiations according to Kedar et al’s research.^[2]^ In addition, infarction, brain tumors, and hemorrhages are the main causes of HH.^[3]^ It means that most causes of HH can be detected as prominent lesions through brain imaging such as brain computed tomography (CT) or magnetic resonance imaging (MRI). However, in rare cases, chronic progressive cerebral venous thrombosis (CVT) occurring in postchiasmal visual pathway also can cause HH, which may not be easily detected on imaging.^[4]^ Because CVT is a rare cerebrovascular disease with nonspecific symptoms,^[5]^ it is important to find out the clinical characteristics and disease progression of patients with ophthalmic symptoms from CVT. Herein, we present a case of delayed diagnosis of CVT due to nonspecific neurological symptoms and nonprominent lesions in a patient with HH. The Daegu Catholic University Hospital Institutional Review Board (IRB) for Human Studies reviewed and approved the protocol (IRB no. CR-23-143). The patient has provided informed consent for the publication of the case.

## 2. Case report

**Figure 1.**
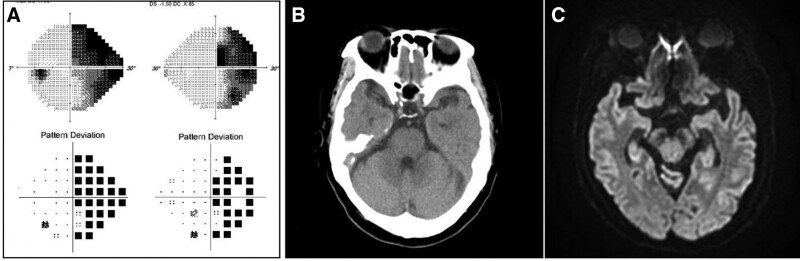
Humphrey visual field (24-2 HVF) testing and brain imaging at the patient’s first visit. (A) Right homonymous hemianopia is noted in the HVF test. (B) No evidence of hemorrhage or mass is seen on brain CT. (C) In brain diffusion MRI, no pathologic lesion is noted in the brain parenchyma. CT = computed tomography, MRI, magnetic resonance imaging.

A 65-year-old woman with history of hypothyroidism and dyslipidemia presented to emergency room with sudden onset headache accompanied by right HH for 1 day. She described headache as a sharp pain persisting on the forehead and occipital area, with nausea and vomiting. Ophthalmological examination revealed a best-corrected visual acuity of 20/20 in both eyes normal intraocular pressure. Her Ishihara test results for color perception were normal and extraocular muscle movements were full without ocular moving pain. Pupillary light reflex examination showed that her pupils were isocoric and reactive with no afferent pupillary defect. Slit-lamp examination revealed no cells in the anterior chamber or vitreous and fundus examination results were unremarkable. Humphrey automated visual field testing revealed right HH, which was more prominent in the upper quadrant (Fig. 1A). Therefore, it was decided to take a brain imaging to find out the brain lesion that may be the cause of HH and headache. However, noncontrast brain CT of her was unremarkable and diffusion MRI of the head showed a normal brain parenchyma without any inflammation or infarction (Fig. 1B,C). Blood sampling was performed to rule out inflammation, infection, and autoimmune causes. Laboratory results showed high levels of thyroid stimulating hormone (6.9 uU/mL) and cholesterol (213 mg/dL), but these were not considered a cause of headache as she had been steadily treated with medication. After confirming the absence of significant brain lesions on brain imaging and conservative treatment for the headache, the patient’s symptoms disappeared. Hence, the patient was discharged and admitted to the outpatient clinic several days later.

**Figure 2.**
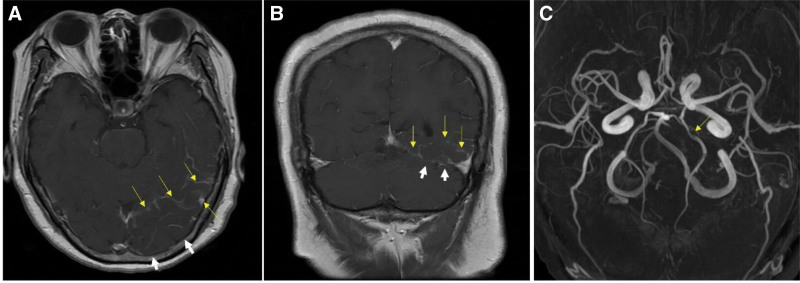
Brain MRI and MRA at the patient’s second visit. Brain MRI reveals gyral swelling in the left temporo-parieto-occipital lobes (white arrow) and sulcal vascular enhancement (yellow arrow) around the swelling in the T1 axial view (A) and T1 coronal view (B). Brain MRA reveals focal stenosis on the left proximal posterior cerebral artery (yellow arrow), which does not seem significant enough to cause homonymous hemianopia. MRA = magnetic resonance angiography, MRI = magnetic resonance imaging.

**Figure 3.**
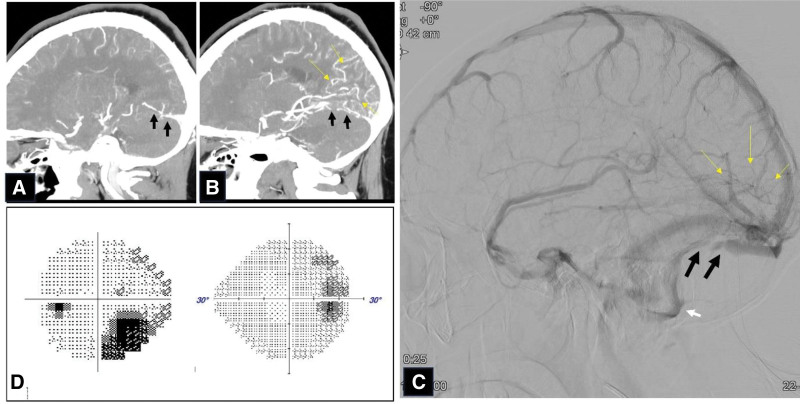
Three-dimensional cerebral CT angiography, 4-vessel angiography following admission, and Humphrey visual field testing following treatment. Three-dimensional cerebral CT angiography reveals contrast well draining through the right transverse sinus (black arrow) (A) and filling defect (black arrow) and superior venous drainage impairment (yellow arrow) (B) on the left transverse sinus. Four-vessel angiography (C) reveals a filling defect of the left transverse sinus (black arrow), indicating the presence of CVT and venous congestion (yellow arrow). However, venous blood flow through the left sigmoid sinus (white arrow) is observed, indicating incomplete occlusion, leading to the decision for medical treatment instead of surgical intervention. Following anticoagulant treatment, the patient’s visual field defect (D) improved significantly compared to the initial right homonymous hemianopia. CT = computed tomography, CVT = cerebral venous thrombosis.

The patient revisited the emergency room 1 day after being discharged owing to worsening headache that had progressed throughout the day. She reported the recurrence of headache accompanied by right HH approximately 3 hours prior to her return. To reassess her condition, the patient underwent brain MRI and magnetic resonance (MR) angiography (MRA) of the head and neck. Sectoral gyral swelling in the occipital lobe with prominent sulcal vascular enhancement and hyperintensity in the left temporo-parieto-occipital lobe were observed, suggestive of focal vasogenic congestion (Fig. 2A,B). In her brain MRA, only focal stenosis on the left proximal posterior cerebral artery was seen that did not seem significant to cause right HH (Fig. 2C). Hospitalization was decided for further examination. To evaluate the impairment of venous drainage, a 3-dimensional cerebral CT angiography was conducted, and imaging revealed a partial filling defect in the left transverse sinus, along with superior venous drainage impairment, suggesting the presence of venous thrombosis in left transverse sinus (Fig. 3A,B). Four-vessel angiography was performed to evaluate the severity of CVT and to determine a treatment (Fig. 3C). Filling defect of her left transverse sinus indicating the presence of CVT was noted. However, venous blood flow through left sigmoid sinus was observed, which meant that there was incomplete occlusion. Additional laboratory tests, including D-dimer, protein C, protein S, and antithrombin levels, were within normal limits. Consequently, the patient was finally diagnosed with thrombosis in the left transverse sinus, which subsequently caused right HH. Because brain edema due to CVT was focal and left transverse sinus thrombosis seems to induce incomplete occlusion, medical treatment was decided instead of surgical treatment such as thrombectomy. Anticoagulant treatment was initiated. Treatment was initiated with intravenous heparin of 25,000 IU/d (Choongwae Pharma Corporation, Seoul, Korea) for 1 week, followed by a transition to oral warfarin administration of 4 mg along with daily subcutaneous low-molecular-weight heparin (Fraxiparine) injection of 5700 IU for 2 weeks. Eventually, the patient was solely managed with warfarin of 10 mg. Three days after treatment, her headache resolved and visual field testing conducted 2 weeks later revealed a definite improvement in the field defect (Fig. 3D). After discharge, the patients were lost to follow-up.

## 3. Discussion

The present case had prominent HH, but the diagnosis of CVT was delayed because of nonspecific neurological symptoms and minimal lesions in the brain parenchyma as observed on brain imaging. Fortunately, after being diagnosed with CVT, the headache and HH symptoms resolved with anticoagulant medication.

CVT is a rare cerebrovascular disease that accounts for 0.5% of all strokes.^[5]^ It typically affects the superior sagittal and transverse sinus more frequently.^[6]^ As CVT presents in various locations, its clinical manifestation also presents various symptoms depending on the location and mimics other disorders. Diagnosing CVT is challenging and takes an average of several days^[7]^ owing to its low incidence and nonspecific symptoms, including headache, nausea, vomiting, and altered consciousness.

Previous reports have highlighted the ophthalmologic symptoms associated with CVT. Thrombosis in the cavernous sinus, which contains all 3 ocular motor nerves and oculosympathetic fibers entering the orbit, can cause various ophthalmic complications, including ocular pain, chemosis, proptosis, and ocular nerve palsy.^[7]^ Additionally, cortical venous sinus thrombosis and dural arteriovenous fistulae can lead to elevated intracranial pressure, resulting in secondary papilledema or abducens palsies.^[8–10]^ Eliseeva et al^[6]^ reported that among 49 patients with CVT, 84.6% with acute and subacute disease onset and all patients with chronic disease onset had papilledema. Complications of visual field defects have also been reported. Tatsuoka et al^[11]^ reported the case of a 28-year-old woman who developed HH and headaches after receiving Infliximab. Hemorrhagic infarction and transverse sinus thrombosis were detected using CT and angiography, respectively. Grabe et al^[12]^ reported that a 20-year-old man with HH, hemiparesis, and hemisensory loss was diagnosed with brain hemorrhage on brain CT and deep cerebral venous thrombosis on MR venography. Unlike previous case reports of HH from CVT, our patient had only HH and headache without any neurological symptoms or specific events, such as trauma or vaccination history, which made it difficult for clinicians to consider CVT as a primary impression.

The key to diagnosing CVT lies in imaging the venous system through MR or CT venography, which may reveal an occluded vessel or intravascular thrombus. However, false positives should be considered when interpreting images that show a physiological hypoplastic transverse sinus in normal situations.^[7]^ Therefore, brain CT and MRI are helpful initial tests because most cases of CVT can be detected as definite lesions, such as ischemic parenchyma, subarachnoid hemorrhage, or signs of edema. However, in the present case, no significant brain lesions were observed when the patient first visited the emergency room. Ulivi et al^[7]^ mentioned that the slow growth of the thrombus and collateralization of venous vessels probably account for the often gradual onset of symptoms, frequently over days, weeks, or even months. In line with Ulivi et al’s report, collateral venous drainage around the left transverse sinus was confirmed in the present case, suggesting that the thrombosis was a chronic progressive lesion rather than an acute development. No definite brain lesions, such as infarction or hemorrhage were observed; only gyral swelling was noted on repeat brain MRI.

Moreover, clinicians would better identify risk factors that can lead to CVT. Approximately 85% of adult patients with CVT have at least one associated risk factor.^[7]^ Key risk factors for CVT include using oral contraceptives, pregnancy, and prothrombotic conditions, such as antiphospholipid antibody syndrome, coagulopathy, or thrombophilia. Our patient did not use oral contraceptives and exhibited no coagulation abnormalities. Therefore, the cause of thrombosis, in this case, was unclear; however, hemodynamic instability, such as dyslipidemia, may have induced the thrombosis.

The treatment guidelines for CVT vary depending on the severity of symptoms, lesions, and disease duration. In general, prompt initiation of rapid anticoagulation therapy with parenteral heparin is recommended as soon as the diagnosis of CVT is confirmed.^[13]^ In cases with acute onset and symptom duration of <15 days, local endovascular thrombolysis in combination with mechanical thrombectomy can be used. Because of relatively minor brain lesions and chronic partial occlusion, our patient received only medical anticoagulant treatment; intravenous heparin injection, subcutaneous low-molecular-weight heparin, and oral warfarin administrations. Following 2 weeks of medical treatment, the headache and HH resolved. This case report has limitations that the patient did not visit the hospital after her symptoms improved thus long-term follow-up observation was not possible.

## 4. Conclusions

In conclusion, when patients present with HH and associated vascular risk factors, even without prominent intracerebral lesions in the postchiasmal visual pathway, CVT should be considered as a differential diagnosis, along with the other common causes, such as cerebral infarction, brain tumor, or intracranial hemorrhage. Furthermore, if the initial eye symptoms persist without improvement, active imaging reexamination is necessary because of the potentially slow progression of CVT.

## Acknowledgments

We would like to thank Editage (www.editage.co.kr) for English language editing.

## Author contributions

**Data curation:** Woo Seok Choi, Donghun Lee.

**Resources:** Woo Seok Choi, Donghun Lee.

**Writing** – **original draft:** Woo Seok Choi, Donghun Lee.

**Investigation:** Sook Hyun Yoon, Donghun Lee.

**Conceptualization:** Donghun Lee.

**Writing** – **review & editing:** Donghun Lee.
